# Prevalence and predictors of suicidal behaviours among primary and secondary school going adolescents in Botswana

**DOI:** 10.1371/journal.pone.0282774

**Published:** 2023-03-14

**Authors:** James Forty, Kannan Navaneetham, Gobopamang Letamo

**Affiliations:** 1 African Institute for Development Policy, Lilongwe, Malawi; 2 Department of Population Studies, University of Botswana, Gaborone, Botswana; King Khalid University, EGYPT

## Abstract

**Background:**

There is a scarcity of studies on the prevalence and predictors of suicide behaviors among primary and secondary school going adolescents aged 10–19 years in Botswana hence, this study would fill that gap.

**Methods:**

This study used cross-sectional secondary data from Botswana Youth Risk Behavior and Biological Surveillance Survey (BYRBBSS), 2010. Multivariable binary logistic regression models were used to investigate the predictors of suicide behaviours.

**Results:**

The study shows that 51.5% of the respondents reported having ever contemplated suicide while 40.1% of the respondents attempted suicide in the last 12 months before the survey. The study found that male learners (AOR = 0.61, 95% CI = 0.44–0.83), learners who were not attacked or threatened or injured by someone (AOR = 0.35, 95% CI = 0.17–0.72), who were not bullied (AOR = 0.22, 95% CI = 0.13–0.39), and who were confident of themselves (AOR = 0.55, 95% CI = 0.39–0.76) were less likely to contemplate suicide. Whereas learners with primary school level education were more likely to contemplate suicide (AOR = 2.12, 95% CI = 1.14–3.95). Males were less likely than their female counterparts to attempt suicide (AOR = 0.68, 95% CI = 0.47–0.97). Regarding attempt, learners who had self-confidence (AOR = 0.35, 95% CI = 0.24–0.50), not being bullied (AOR = 0.20, 95% CI = 0.11–0.35), not being attacked or threatened or injured by someone (AOR = 0.35, 95% CI = 0.18–0.69), not engaging in a physical fight that led to injury (AOR = 0.34, 95% CI = 0.19–0.61) were less likely to attempt suicide. Whereas being at primary school (AOR = 5.29, 95% CI = 2.58–10.86), and missing classes once or more in a week (AOR = 1.70, 95% CI = 1.05–2.76) were associated with increased likelihood of suicide attempt.

**Conclusion:**

The study shows that suicide behaviours as big challenges in Botswana among primary and secondary school going adolescents aged 10–19 years. Thus, the study recommends policy interventions aiming at including education on peer bullying or fighting or attack at primary and secondary education levels if not already in existence. There should also be interventions aiming at educating guardians and teachers on consequences of bullying or fighting so that they should consistently remind their children not to bully and for them to open up if they are being bullied. The study further recommends that schools and communities at large should have a psychosocial system for bullying or fighting reporting, follow-up, and appropriate corrective interventions for the offenders. There should also be self-confidence instilling education as well as sex/gender-specific interventions for instance girls can be given platform (private if necessary) to express peculiar problems to them that need specific help.

## Introduction

Suicidal behaviors present a major challenge to public health globally and in particular for adolescents and youth aged 10–24 years [1]. Suicidal behaviour is defined as a range of behaviours that include thinking about suicide (or ideation), planning for suicide, attempting suicide, and suicide itself [1]. World Health Organization’s mortality data, between the period of 2010 to 2016, estimated a suicide rate of 3.8/100000 among adolescents aged 10–19 years [2]. Although no literature is found about the suicide rate among adolescents especially primary and secondary going learners aged 10–19 years in Botswana, the general population’s suicide rate in 2016 was estimated at 9.70 deaths per 100000 populations [3]. Nevertheless, the study done on undergraduate University of Botswana students found that 47.5% of the respondents contemplated about suicide and 28.7% attempted suicide [4]. About the prevalence, studies, especially in sub-Sahara Africa, have found some variations in suicide ideation among school-going adolescents thus, in Zambia 31.3% (males = 31.1%; females = 31.4%) [5]; while in Uganda 21.6% (21.3% males and 23.5% females) [6]. On the other hand, the prevalence of suicidal ideation attempt in Ethiopia were 20.5% [males = 17.6%, and females = 23.8%] and 12.5% [males = 11.1%, females = 14.0%] respectively [7].

Meanwhile, studies in Sub-Sahara Africa have found that suicidal behaviours (especially among the adolescents) are associated with not having close friends, not having an understanding with parents, age of the adolescent, heavy alcohol intake behaviour, illicit drug abuse, smoking, loneliness, hopelessness, sadness, anxiety, poor class results, marital or family conflict, lack of social support, parental neglect, trading sex for food or shelter or money, a victim of bullying, HIV positive, physical fights or attacks, food insecurity, sex, sexual orientation (gay/lesbian/bisexual), missing classes and having a family history of suicide [7–11]. Although the factors associated with suicide behaviors seem to be similar in many sub-Sahara countries, it is necessary to do research peculiar to the contextual setting where such study has never done for the sake of evidence informed policy and programming. Thus, this study is necessary since studies are scarce especially in Botswana with focus on suicidal ideation and attempt for the adolescents aged 10–19 years especially those at primary and secondary school levels. Additionally, there is also scarcity of similar studies focusing on prevalence of suicide behaviors. Therefore, the main aim of this study is to establish the prevalence and predictors of suicide behaviours (suicide ideation and attempt) among primary and secondary school-going adolescents aged 10–19 years in Botswana.

## Methods

### Ethical approval

The study protocol and questionnaire were reviewed and approved by Botswana Ethical Committee and Botswana Human Resource Development Council reviewed and approved the content and language of the informed consent letters and forms. The study also sought ethical clearance through the health research unit to do the research. Parental/guardian consent was sought and learners’ assent was obtained before being selected to participate or volunteer to take part in the study. Thus, informed consent was obtained from all participants or from a parent and/or legal guardian in case the participants were aged under 16 years. The Research Assistants collected all letters sent to parents or guardians and verified that they were signed. Respondents were not coerced to take part in the study; all efforts were made to avoid deception or making false promises to woe participants to agree to be part of the study. Anonymity or Confidentiality of all information providers was assured to protect their individual and personal identities. Names or other personal identifiers were not recorded in any survey instruments. All survey records were marked with an automatically generated questionnaire identification number. Because of the sensitivity of some of the questions, some reactions from the learners were expected hence class teachers were nearby to assist. Therefore, all methods were performed in accordance with the relevant guidelines and regulations.

Prior to the issuance of the informed consent, the Chief Education Officers discussed the survey to school heads, teachers, and parents through parent and teacher meetings. Then, the written informed consent was sought from learners’ parents. Parents’ consents were sought first before the random sampling of the respondents to help with the sampling from among the students whose parents have given consents thus, mitigating non-response rate. However, the explanation was given to the students pertaining the study for them to also make informed decision regarding their participation in the survey. This ensured a negligible non-response rate among the sampled. Moreover, the sampled learners that were absent the day of the survey were still completing the survey and they were assured of their privacy. Allowing learners who were absent on the day of data collection to take the survey at a later date increased learners’ response rates.

### Study design and data source

This study used cross-sectional secondary data from Botswana Youth Risk Behavior and Biological Surveillance Survey (BYRBBSS), which was collected in 2010. The sampling frame consisted of all public upper primary (Standard 5–7) and secondary (Form 1–5) schools in Botswana from which a sample size of 3863 was taken. The survey used a two-stage cluster sample design to generate a representative sample of students aged 10–19 years in public schools across the country. Thus, each district, of the 15 Ministry of Education’s districts, employed a two-stage sample design to produce representative samples of learners. The primary sampling unit was the school. Five schools per district were selected for participation in the survey using probability proportional to size sampling methods based on total enrolment at the school. The sampling frame was developed using a master list of schools and 2007 enrolment data maintained by the Ministry of Education. The secondary sampling unit was the classroom. Two classes per school needed were selected randomly.

### Study variables and measurements

#### Dependent variables

The outcome variables of this study are suicide ideation and suicide attempt. Suicide ideation was derived from a question that asked the learners “during the past 12 months, how many times did you think about suicide”. Likewise, suicide attempt was derived from the question that asked the learners “during the past 12 months, how many times did you attempt suicide”. Respondents who reported to have thought of suicide or attempted suicide were given a code of **1** and a code of **0** if reported to have never thought of suicide or attempted suicide.

#### Independent variables

The independent factors are alcohol, cigarette and other illicit drugs, frequency of going hungry, frequency missing classes, physical fights or attacks, alcohol, cigarette and other illicit drugs frequency of being bullied, having fights resulting in injuries, and being emotionally abused by family or teacher, sex of an individual, age, school level, lack of self-confidence, and place of residence. Respondents who stated that they consume alcohol or smoke cigarette or take other illicit drugs were given a label of ‘yes’ otherwise the label given was ‘no’. Frequency of missing classes was measured as whether it was once/more per week, or once a month, or never. Frequency of going was measured as to whether was never, or sometime, or always. Frequency of being bullied in the past 30 days prior to the survey was measured as whether it was 1 to 5 days, or more than 5 days, not bullied. The variables thus, Emotional abuse by family or teacher or being attacked/injured/threatened by someone in the past 30 days prior to the survey were given labels thus, ‘no’, ‘once’ and ‘more than once’. These same labels (no, once, more than once) were also used for the variable ‘physical fights that led to injuries in the past 12 months prior to the survey’. Either the respondent was ‘male’ or ‘female’ were the labels given in relation to the variable ‘sex of the respondent. Age of the respondent was categorized as 10 to 12 years, 13 to 15 years, and 16 to 19 years. School level was categorized as primary, junior secondary, and senior secondary. Lack of confidence was measure as to whether the respondent had confidence thus, a ‘yes’ label if not the ‘no’ label. Place of residence was given categories of either urban or rural. These variables are used in this study because they were found associated with suicide ideation or attempt by other studies from sub-Sahara Africa [7–11].

### Data analysis

Before data analysis, the data was cleaned. Furthermore, some variables (age, missing classes, frequency of going hungry, frequency of being bullied, physical fights with associated injuries, threats or attacks, and emotional abuse by teachers or family) were recoded to meet the objectives of the study. Age was grouped into three age groups thus 10–12 years, 13–15 years, and 16–19 years for easy analysis. Moreover, the preliminary analysis showed that majority of those in age group 10–12 years were at primary school level while those in age group 13–15 years and 16–19 years majority of them were at junior and senior secondary levels respectively. Regarding frequency of missing school, there were many categories, which based on preliminary results some categories had almost 0% thus, we had to recode (come up with categories) which could give plausible percentage outputs thus, one or more per week, once in a month, and never. The just given reasons for the preceding recoded variable hold true for other variables thus, frequency of going hungry, frequency of being bullied, physical fights with associated injuries, threats or attacks, and emotional abuse by teachers or family. Hence, frequency of going hungry was recoded into three categories as never, sometimes, and always. Frequency of being bullied was recoded into three categories thus, not bullied, 1 to 5 days and more than 5 days. Physical fights with associated injuries in the past 12 months prior to the survey, and being threatened/attacked/injured by someone in the past 30 days prior to the survey were recoded into three categories thus, no, once, and more than once. Likewise, emotional abuse by the family and emotional abuse by the teacher were also recoded as no, once, and more than once.

Suicide ideation and attempt were analysed separately. Descriptive statistics were used to come up with summarized univariate data and the results were presented as proportions (%). At the bivariate level, each independent variable was cross-tabulated with the dependent variable, and Pearson Chi-Square was used to establish the significant association between each independent variable and dependent variable. Variables that were found significant at the bivariate level were used for regression analysis. Binary logistic regression analysis was used to identify predictors of suicide ideation and suicide attempt. Adjusted odds ratios and their 95% confidence intervals (95% CI) were estimated. All statistical analyses were performed using Statistical Package for the Social Sciences (SPSS, version 25) and the statistical significance was set at a P-value of less than 0.05.

The logistic regression equation of multiple predictors takes the following form;

Logit(y)=ln(p1‐p)=α+β1X1+……+βkXk_.

Where P is the probability of the interested outcome and Xs are the explanatory variables. The parameters of the logistic regression are α and β.

## Results

### Sample description

The characteristics of the study population are presented in Table 1. About half (51.5%) of the respondents reported having ever contemplated suicide while two-fifth (40.1%) of the respondents attempted suicide in the last 12 months before the survey. Concerning explanatory variables, most of the respondents were females (55.7%), of age group 13–15 years (41.3%), living in rural areas (83.9%), and of primary school level (48.7%). Furthermore, more than one-third of the respondents reported missing classes (34.4%), more than half reported going hungry (53.2%), more than one-tenth reported consuming alcohol (17.3%), about one-fifth reported smoking a cigarette (19.2%), and more than one-fifth reported taking drugs (22.2%). Likewise, in the past 30 days before the survey, close to half of the respondents reported to have experienced bullying (45.3%), more than half reported of emotional abuse from teachers (68.7%) or family (65.9%) or attacks or threats injured by someone (70.7%). Close to one-third of the respondents (31.3%) reported to have had fights that led to an injury. More than one-third of the respondents (37.0%) reported that they were confident about themselves.

**Table 1:** Sample characteristics of primary and secondary schools’ learners in Botswana aged 10–19 years at the time of the survey, BYRBBSS (N = 4289).

Variable	N	%
***Sex***		
Male	1710	44.3
Female	2153	55.7
***Age group***		
10–12 years	1214	31.5
13–15 years	1593	41.3
16–19 years	1052	27.3
***Place of residence***		
Urban	688	16.1
Rural	3589	83.9
***Class level***		
Primary	1880	48.7
Junior secondary	1500	38.9
Senior secondary	481	12.5
***Frequency of missing school***		
Once or more per week	645	19.1
Once a month	517	15.3
Never	2213	65.6
***Alcohol consumption***		
Yes	635	17.3
No	3026	82.7
***Cigarette smoking***		
Yes	703	19.2
No	2952	80.8
***Drug taking***		
Yes	752	22.2
No	2641	77.8
***Frequency of going hungry***		
Never	1581	46.8
Sometimes	1251	37.0
Always	549	16.2
***Frequency of being bullied the past 30 days***		
Not bullied	1750	54.7
1–5 days	868	27.1
More than 5 days	581	18.2
***Emotionally abused by teachers the past 30 days***		
No	2291	67.8
Once	552	16.3
More than once	538	15.9
***Emotionally abused by family the past 30 days***		
No	2219	65.9
Once	578	17.2
More than once	568	16.9
***Attacked or threatened or injured by someone the past 30 days***		
No	2334	70.7
Once	463	14.0
More than once	504	15.3
***Physical fight that led to injury in the past 12 months***		
No	2270	68.7
Once	446	13.5
More than once	590	17.8
***Self-confidence***		
Yes	2008	63.0
No	1181	37.0
***Contemplated suicide the past 12 months***		
No	1504	48.5
Yes	1599	51.5
***Attempted suicide the past 12 months***		
No	1871	59.9
Yes	1250	40.1
**Total**	**3863**	**100**

### Association between explanatory variables and suicidal-behaviours

#### Suicide ideation

Table 2 shows the bivariate association between explanatory variables and suicidal behavior. The results in Table 2 show that suicide ideation was significantly associated with all variables except the sex and place of residence of the respondent. The results showed a decreasing pattern of suicide ideation with an increase in age and school level. On the other hand, the results show increased pattern of suicide ideation with increased frequency of missing classes or going hungry or being bullied or emotionally abused or threatened/attacked or fight that led to the injury. Regarding the frequency of going hungry, the pattern shows that the respondents who were always hungry had the highest percentage that contemplated suicide followed by the respondents who were hungry sometimes and the respondents who were never hungry showed the lowest suicide ideation. Similarly, the results also show increasing patterns of suicide ideation with increasing frequency of experiencing bully or attack or threat or injured by someone or emotionally abused by the teachers or family in the past 30 days preceding the survey. Likewise, the pattern of increasing suicide ideation holds with increasing fights that led to injury in the past 12 months preceding the survey. Meanwhile, the respondents who were drinking alcohol, smoking cigarettes, taking drugs had higher percentages of respondents that contemplated about suicide than the percentages of respondents who were not.

**Table 2:** Bivariate associations (based on Chi-squared tests) between explanatory variables and each suicidal-behaviour.

Variable	*Suicide ideation*		*Suicide attempt*	
	N	%	N	%
***Sex***				
Male	724	53.2	582	42.6
Female	875	50.2	668	38.1
p-value	0.24		0.010	
***Age group***				
10–12 years	568	64.4	526	58.8
13–15 years	697	54.0	543	42.1
16–19 years	333	35.9	180	19.3
p-value	0.000		0.000	
***Place of residence***				
Urban	245	47.8	174	33.2
Rural	1353	52.3	1075	41.4
p-value	0.12		0.000	
***Class level***				
Primary	945	68.2	878	62.9
Junior secondary	501	39.5	308	24.2
Senior secondary	153	34.1	64	14.3
p-value	0.000		0.000	
***Frequency of missing school***				
Once or more a week	401	74.0	367	68.9
Once a month	280	68.0	247	60.4
Never	752	39.8	482	25.3
p-value	0.000		0.000	
***Alcohol consumption***				
Yes	324	55.5	210	37.9
No	1234	50.0	992	39.7
p-value	0.018		0.09	
***Cigarette smoking***				
Yes	367	60.2	264	43.2
No	1172	48.5	934	38.3
p-value	0.000		0.026	
***Drugs taking***				
Yes	399	62.5	345	53.6
No	1047	47.2	781	34.9
p-value	0.000		0.000	
***Frequency of going hungry***				
Never	559	40.1	397	28.5
Sometimes	606	56.3	458	41.9
Always	339	71.7	306	64.2
p-value	0.000		0.000	
***Frequency of being bullied the past 30 days***				
Not bullied	503	31.3	284	17.5
1–5 days	568	70.4	475	58.2
More than 5 days	414	83.8	405	80.4
p-value	0.000		0.000	
***Emotionally abused by teachers the past 30 days***				
No	808	41.1	582	29.6
Once	293	58.3	228	43.9
More than once	383	82.5	351	73.7
p-value	0.000		0.000	
***Emotionally abused by family the past 30 days***				
No	767	40.7	576	30.3
Once	306	58.3	214	40.6
More than once	398	78.8	360	70.2
p-value	0.000		0.000	
***Attacked or threatened or injured by someone the past 30 days***				
No	798	38.4	512	24.4
Once	284	69.1	251	60.0
More than once	385	86.3	376	83.0
p-value	0.000		0.000	
***Physical fight that led to injury in the past 12 months***				
No	741	36.7	439	21.6
Once	286	73.3	261	64.9
More than once	433	84.4	430	83.0
p-value	0.000		0.000	
***Self-confidence***				
Yes	718	40.5	492	27.5
No	717	69.8	633	61.0
p-value	0.000		0.000	

Statistical significance set at p< 0.05; ns, not significant

#### Suicide attempt

The results in Table 2 indicate that suicide attempt is significantly associated with all variables except alcohol consumption. The results reveal that the male respondents who were living in rural areas had higher percentages of suicide attempts than the respondents who were not. Furthermore, the results show the decreasing pattern of suicide attempts with increase in age or school level. The results also show an increase in suicide attempt with increased frequency of missing classes, increased frequency of going hungry, and frequency of being bullied or emotionally abused or threatened/attacked or injured by someone in the past 30 days before the survey or as well as increased frequency of fights that led to the injury in the past 12 months preceding the survey. A high percentage of the respondents who were smoking cigarettes and taking drugs were associated with an increased chance of attempted suicide than the respondents who were not. On the other hand, the respondents who had confidence in themselves had lower percentages of suicide attempts than the respondents who had not.

### Predictors of suicide behaviours among learners aged 10–19 years

#### Suicide ideation

Table 3 shows the odds ratios of the explanatory variables, with the adjusted model, predicting the likelihood of suicide ideation. The results show that suicide ideation was significantly associated with the sex of the learner, class level, frequency of being bullied, attacked, or threatened, or injured by someone in the past 30 days, and self-confidence. Compared to the female learners, male learners were less likely to contemplate suicide (AOR = 0.61, 95% CI = 0.44–0.83). Learners with primary school level education were more likely to contemplate suicide than learners with secondary level of education (AOR = 2.12, 95% CI = 1.14–3.95). Learners who were not bullied were less likely to contemplate suicide than learners who were bullied for more than five days (AOR = 0.22, 95% CI = 0.13–0.39). Learners who were not attacked or threatened or injured by someone in the past 30 days before the survey were less likely to think about committing suicide than those who were attacked or threatened or injured by someone more than once (AOR = 0.35, 95% CI = 0.17–0.72). Learners who were confident of themselves were less likely to contemplate suicide than those who were not confident of themselves (AOR = 0.55, 95% CI = 0.39–0.76).

**Table 3:** Adjusted odds ratios showing the likelihood of suicide behaviours among learners aged 10–19 years.

Variable	*Suicide ideation*		*Suicide attempt*	
	Adjusted model		Adjusted model	
	Exp B	95% C.I.	Exp B	95% C.I.
***Sex***				
Male	0.61*	0.44–0.83	0.68*	0.47–0.97
Female	1.00		1.00	
***Place of residence***				
Urban	1.01	0.68–1.45	1.12	0.71–1.76
Rural	1.00		1.00	
***Age group***				
10–12 years	1.57	0.86–2.90	1.88	0.97–3.62
13–15 years	1.49	0.97–2.27	1.21	0.74–1.97
16–19 years	1.00		1.00	
***Class level***				
Primary	2.12*	1.14–3.95	5.29*	2.58–10.86
Junior secondary	0.89	0.56–1.39	1.68	0.94–3.00
Senior secondary	1.00		1.00	
***Frequency of missing school***				
Once or more a week	1.28	0.80–2.05	1.70*	1.05–2.76
Once a month	0.99	0.62–1.59	1.29	0.78–2.13
Never	1.00		1.00	
***Alcohol consumption***				
Yes	1.09	0.75–1.60	0.81	0.52–1.28
No	1.00		1.00	
***Cigarette smoking***				
Yes	1.36	0.93–1.99	1.23	0.79–1.90
No	1.00		1.00	
***Drugs taking***				
Yes	1.21	0.81–1.80	1.29	0.84–1.99
No	1.00		1.00	
***Frequency of going hungry***				
Never	0.97	0.59–1.62	1.12	0.65–1.94
Sometimes	1.54	0.92–2.57	1.34	0.78–1.32
Always	1.00		1.00	
***Frequency of being bullied the past 30 days***				
Not bullied	0.22*	0.13–0.39	0.20*	0.11–0.35
1–5 days	0.63	0.35–1.11	0.38*	0.21–0.67
More than 5 days	1.00		1.00	
***Emotionally abused by teachers the past 30 days***				
No	0.76	0.45–1.29	0.78	0.45–1.35
Once	0.87	0.49–1.55	1.00	0.55–1.83
More than once	1.00		1.00	
***Emotionally abused by family the past 30 days***				
No	0.63	0.38–1.03	1.00	0.58–1.72
Once	1.18	0.49–1.55	1.17	0.65–2.11
More than once	1.00		1.00	
***Attacked or threatened or injured by someone the past 30 days***				
No	0.35*	0.17–0.72	0.35*	0.18–0.69
Once	0.48	0.23–1.00	0.54	0.27–1.09
More than once	1.00		1.00	
***Physical fight that led to injury in the past 12 months***				
No	0.63	0.34–1.14	0.34*	0.19–0.61
Once	1.13	0.58–2.19	0.67	0.36–1.27
More than once	1.00		1.00	
***Self-confidence***				
Yes	0.55*	0.39–0.76	0.35*	0.24–0.50
No	1.00		1.00	

***p<0.05

Other variables such as age group, place of residence, frequency of missing classes, frequency of going hungry, alcohol consumption, cigarette smoking, taking drugs, emotional abuse by teachers or family, and physical fight that led to injury were found not to be predictors of suicide ideation.

#### Suicide attempt

Table 3 also shows that being male, being confident about oneself, experiencing less bullying or attack or threat or injured by someone in the past 30 days as well as the physical fight that led to injury in the past 12 months were significantly associated with decreased odds of a suicide attempt. Males were less likely than their female counterparts to attempt suicide (AOR = 0.68, 95% CI = 0.47–0.97). Self-confidence reduces the likelihood of considering suicide attempt (AOR = 0.35, 95% CI = 0.24–0.50). Not being bullied also reduces the likelihood of attempting suicide compared to those who were bullied (AOR = 0.20, 95% CI = 0.11–0.35). Not being attacked or threatened or injured by someone was associated with less likelihood of suicide attempt (AOR = 0.35, 95% CI = 0.18–0.69). Not engaging in a physical fight that led to injury was associated with less likelihood of suicide attempt compared to those who engaged in physical fight (AOR = 0.34, 95% CI = 0.19–0.61).

However, having primary level education and an increased frequency of missing classes were significantly associated with the likelihood of a suicide attempt. Having a primary school level education was associated with increased odds of suicide attempt (AOR = 5.29, 95% CI = 2.58–10.86) compared to those with secondary level education. Missing classes one or more in a week were associated with increased likelihood of suicide attempt compared to never missing classes education (AOR = 1.70, 95% CI = 1.05–2.76).

Other variables such as age group, place of residence, frequency of going hungry, alcohol consumption, cigarette smoking, taking drugs, emotional abuse by teachers or family were found not to be predictors of suicide attempt.

## Discussion

This study looked into the prevalence and predictors of suicide behaviors suicide among primary and secondary school-going adolescents aged 10–19 years in Botswana. The study used the data from cross-sectional data thus Botswana Youth Risk Behavior and Biological Surveillance Survey (BYRBBSS), 2010. Regarding the prevalence, the findings show that majority (51.5%) of the respondents contemplated about suicide of which 53.2% were males and 50.2% were females. The findings show that the prevalence of suicide ideation is higher in Botswana among the adolescents aged 10–19 years especially the learners of primary and secondary schools compared to other sub-Saharan countries thus, in Zambia (31.3% of which males = 31.1% and females = 31.4%) [4]; while in Uganda (21.6% of which 21.3% males and 23.5% females) [5]. About suicide attempts, 40.1% of the study’s respondents attempted suicide of which 42.6% were males and 38.1% were females. Similarly, the prevalence of suicide attempts is higher in Botswana compared to other African nations especially Ethiopia (12.5% of which males = 11.1% and females = 14.0%) [6].

The higher prevalence in suicide behaviours may be attributed to the found predictors. The adjusted logistic regression models have predicted the net association each variable has on outcome variables. The predictors of suicide ideation and attempt are increased frequency of being bullied or being attacked or threatened or injured by someone, sex of the learner, class level, and self-confidence. These findings are consistent with other findings of the same target population thus, in Philippines [8], and sub-Sahara Africa thus Kampala in Uganda, Benin and Ethiopia [7, 10, 11]. Meanwhile, there is a lack of evidence from the findings of the reviewed literature that explicitly contradict the aforementioned predictors of suicide ideation and attempt. The findings also show unexpected results thus, variables that are found not to be predictors of suicide ideation are found to be associated with suicide attempt. These variables are frequency of missing classes and physical fights that led to the injury. It is unexpected because suicide ideation precedes suicide attempts. Nonetheless, such unexpected results can be explained as due to the allowable moderate and inevitable collinearity effect which may distort some variables [12]. Another possible explanation according to available evidence from high-income countries could be that young people tend to act with impulsivity which is argued that can facilitate the onset of suicidal attempts without prior suicidal ideation [10, 13, 14].

There may be risk and protective factors that were not included in the primary research hence, have not been used in this study but may have moderated the association between the variables found significant and suicidal behaviors under consideration. Thus, studies have found the following risk and protective factors to be consistently associated with suicide attempt or/and attempt [15–17]. The risk factors include but not limited to presence of a mental disorder, family history of mental disorder, previous attempts, and psychological factors (hopelessness, impulsivity, cognitive rigidity). The protective factors include but are not limited to problem-solving ability, social-emotional skills, limited access to the means of suicide, cultural and religious beliefs that discourage suicide, and social and family/school/community support or safety [15–18]. Risk and protective factors should not be ignored in understanding, evaluating and intervening in suicidal behaviour.

Other variables such as the age of the respondent, place of residence, frequency of going hungry, alcohol consumption, cigarette smoking, taking drugs, emotional abuse by teachers or family were not found as predictors of suicide ideation and attempt. However, these findings are not consistent with other studies that found all variables, except age groups and urban-rural residence, to be significantly associated with suicide ideation and attempt [7, 8, 10]. Hence, despite these factors may not have been found significant predictors of suicide behaviours in Botswana among the target population, their relevance must not be ignored. About age, although is found not to be significant but can easily be linked to class level as most learners in higher class levels are more likely to be older than learners in lower class levels.

The non-significance of the other stated variables is expected in the context of Botswana. For instance, to go hungry, Botswana is one of the few countries in Africa that fully funds the social protection programs out of its resources including in-kind distribution of food (e.g. school feeding programs, Vulnerable Groups Feeding Program) [19]. Hence, the frequency of going hungry may not reach the point of hopelessness which may lead to suicidal behavior as is argued that suicide ideation is the result of hopelessness due to what would be perceived as endless pain [20]. Regarding alcohol consumption, this is an accepted norm or behavior in Botswana especially considering that legislation aimed at addressing the problem of excessive consumption of alcohol in the country was enacted [21]. On another hand, a study in Botswana found among other reasons that smoking is associated with having seen someone smoking in films or social media, self-image misconceptions, and perceived prevalence of smoking among peers and family smoking [22]. This suggests that smoking (which can be extrapolated to drug abuse) among the respondents may be for pleasure, necessitated by misconceptions and perceptions of the learned behavior of exposure, hence may not be associated with their suicide behaviours. Otherwise, lack of significance of family or teacher’s emotional abuse may suggest that at least most families and teachers are well informed that emotional abuse can affect children negatively hence, they desist from abusing them emotionally.

### Limitations

The study has its limitations. The information was collected through self-reporting, which can distort the accuracy of results through social desirability. For instance, the higher proportions of respondents who attempted suicide was not expected. Thus, that can be explained as social desirability possibility especially considering that at least majority of the respondents are young ones aged 15 years and below (10–12 years is 31.5% and 12–15 years is 41.3%) but it could also be a problem of differentiating between suicide ideation and attempt. The latter, reaffirm the limitation of using secondary data as this study only assumes that the primary research ensured that the respondents knew the difference between suicide ideation and attempt. This also calls for supplementary questions to probe and ascertain if respondents (especially if they are children) know in this case ‘suicide attempt’ other than just asking if they had attempted suicide for a certain specific period before the survey. Another limitation in relation to the use of secondary data is that some variables of interest were not included especially pertaining risk and protective factors. Nonetheless, such factors have been included in the discussion as possible moderating factors of the association between the significant explanatory variables and the outcome variables. Another limitation is that this study has used dataset of 2010 as there is no recent dataset. There may be some changes over the years, but may not be that significant as behaviours changes gradually unless in presence of interventions to influence the rate of change. Thus, validating the use of the available dataset especially considering that is only available option.

## Conclusion

Although the causality cannot be ascertained due to cross-sectional nature of the study, the study has shown that increased frequency of being bullied, missing school classes, being attacked or threatened or injured by someone, sex of learner, class level and self-confidence as the predictors of suicide ideation and attempt. Therefore, for policy considerations, this study recommends the inclusion of curriculum focusing on bullying or peer fighting or attack and their associated consequences at primary and secondary education levels. Additionally, the study recommends interventions aiming at educating guardians and teachers on the consequences of bullying or fighting so that they should consistently remind their children not to bully and they should open up if bullied. The study further recommends that schools and communities at large should have a psychosocial support system for bullying or fighting reporting, follow-up, and appropriate corrective interventions for the offenders. Moreover, the psychosocial support system should also consist of self-confidence instilling education as well as sex/gender-specific interventions for instance girls can be given a platform (private if necessary) to express peculiar problems to them that need specific help.
